# Activation of SIRT6 by DNA hypomethylating agents and clinical consequences on combination therapy in leukemia

**DOI:** 10.1038/s41598-020-67170-8

**Published:** 2020-06-25

**Authors:** Hetty E. Carraway, Sridhar A. Malkaram, Yana Cen, Aymen Shatnawi, Jun Fan, Hamdy E. A. Ali, Zakaria Y. Abd Elmageed, Thomm Buttolph, James Denvir, Donald A. Primerano, Tamer E. Fandy

**Affiliations:** 1 Taussig Cancer Institute, Cleveland Clinic, Cleveland, OH USA; 2 Department of Biology, West Virginia State University, Institute, WV USA; 3 Department of Pharmaceutical Sciences, Albany College of Pharmacy, Colchester, VT USA; 4 Department of Pharmaceutical & Administrative Sciences, University of Charleston, Charleston, WV USA; 5 Department of Biomedical Sciences, Marshall University, Huntington, WV USA; 6 Department of Pharmaceutical Sciences, Texas A&M University, Kingsville, TX USA; 7 Department of Neurological Sciences, University of Vermont, Burlington, VT USA; 8 Present Address: Department of Medicinal Chemistry, Virginia Commonwealth University, Richmond, VA 23219 USA

**Keywords:** Acute myeloid leukaemia, Mechanisms of disease, Haematological cancer

## Abstract

The FDA-approved DNA hypomethylating agents (DHAs) like 5-azacytidine (5AC) and decitabine (DAC) demonstrate efficacy in the treatment of hematologic malignancies. Despite previous reports that showed histone acetylation changes upon using these agents, the exact mechanism underpinning these changes is unknown. In this study, we investigated the relative potency of the nucleoside analogs and non-nucleoside analogs DHAs on DNA methylation reversal using DNA pyrosequencing. Additionally, we screened their effect on the enzymatic activity of the histone deacetylase sirtuin family (SIRT1, SIRT2, SIRT3, SIRT5 and SIRT6) using both recombinant enzymes and nuclear lysates from leukemia cells. The nucleoside analogs (DAC, 5AC and zebularine) were the most potent DHAs and increased the enzymatic activity of SIRT6 without showing any significant increase in other sirtuin isoforms. ChIP-Seq analysis of bone marrow cells derived from six acute myeloid leukemia (AML) patients and treated with the nucleoside analog DAC induced genome-wide acetylation changes in H3K9, the physiological substrate for SIRT6. Data pooling from the six patients showed significant acetylation changes in 187 gene loci at different chromosomal regions including promoters, coding exons, introns and distal intergenic regions. Signaling pathway analysis showed that H3K9 acetylation changes are linked to AML-relevant signaling pathways like EGF/EGFR and Wnt/Hedgehog/Notch. To our knowledge, this is the first report to identify the nucleoside analogs DHAs as activators of SIRT6. Our findings provide a rationale against the combination of the nucleoside analogs DHAs with SIRT6 inhibitors or chemotherapeutic agents in AML due to the role of SIRT6 in maintaining genome integrity and DNA repair.

## Introduction

DNA hypomethylating agents (DHAs) comprise a chemically diverse group of drugs that reverse cytosine DNA methylation and induce re-expression of epigenetically silenced genes. The cytosine nucleoside analogs 5-aza-2’-deoxycytidine (decitabine or DAC) and 5-azacytidine (5AC) were among the first used DHAs and are FDA-approved for treatment of myelodysplastic syndrome (MDS)^1–3^. Both drugs compete with cytosine for DNA incorporation with consequent trapping and degradation of DNA methyltransferase (DNMT) enzymes and inhibition of DNA methylation^4^. Zebularine, another nucleoside analog, was developed to overcome the oral instability of both drugs^5^. Unfortunately, its rapid metabolism was an obstacle in its clinical development^6^. Further chemical modifications of decitabine resulted in guadecitabine (SGI-110), which is a dinucleotide antimetabolite of decitabine with the advantage of being resistant to cytidine deaminase metabolism^7^.

The non-nucleoside DHAs comprise another chemically diverse group of compounds. Hydralazine and procainamide are examples of non-nucleoside DHAs that do not incorporate into DNA but inhibit the enzymatic activity of DNMT^4^. Unfortunately, the need to use high and clinically non-relevant doses of these drugs blocked their clinical use as DHAs. Other small molecule drugs like RG-108 and 6-thioguanine demonstrated a DNA hypomethylating effect through inhibition or degradation of DNMT enzymes, respectively^8,9^. Furthermore, several drugs of natural origin demonstrated anticancer and chemopreventive effect due to their DNA hypomethylating effect. The phytochemicals epigallocatechin gallate (EGCG) and Phenethyl isothiocyanate (PEITC) are examples of these natural products and both were shown to inhibit DNMT enzymes^10,11^.

A common feature of all these DHAs is their multiple biological targets. For instance, the nucleoside analogs were shown to modulate histone acetylation^12,13^, histone methylation^14^, induce intracellular reactive oxygen species (ROS) accumulation^15^ and DNA damage^16^. The non-nucleoside analogs were also shown to induce histone modifications and ROS generation^17^. However, the mechanism by which the nucleoside analogs DHAs induce histone acetylation changes is unclear and speculated to be mediated through an indirect mechanism that does not involve direct activation or inhibition of histone acetyltransferase (HAT) or histone deacetylase (HDAC) enzymes.

In this study, we compared the relative potency of different DHAs in reversing DNA methylation and screened their activity against the NAD^+^-dependent HDAC sirtuin isoforms (SIRT1, SIRT2, SIRT3, SIRT5 and SIRT6). The nucleoside analogs DAC, 5AC and zebularine were more potent than the non-nucleoside DHAs in reversing DNA methylation. They significantly increased the enzymatic activity of SIRT6 in both recombinant enzyme assay and in nuclear lysates from leukemia cells without increasing the transcription or protein expression of SIRT6. DAC induced genome-wide changes in the acetylation of H3K9, the physiological substrate of SIRT6. Our results identify a novel target for the cytosine nucleoside analogs DHAs that will impact the rational use of these drugs in combination therapy.

## Results

### The cytosine nucleoside analogs are the most potent DHA

**Figure 1:**
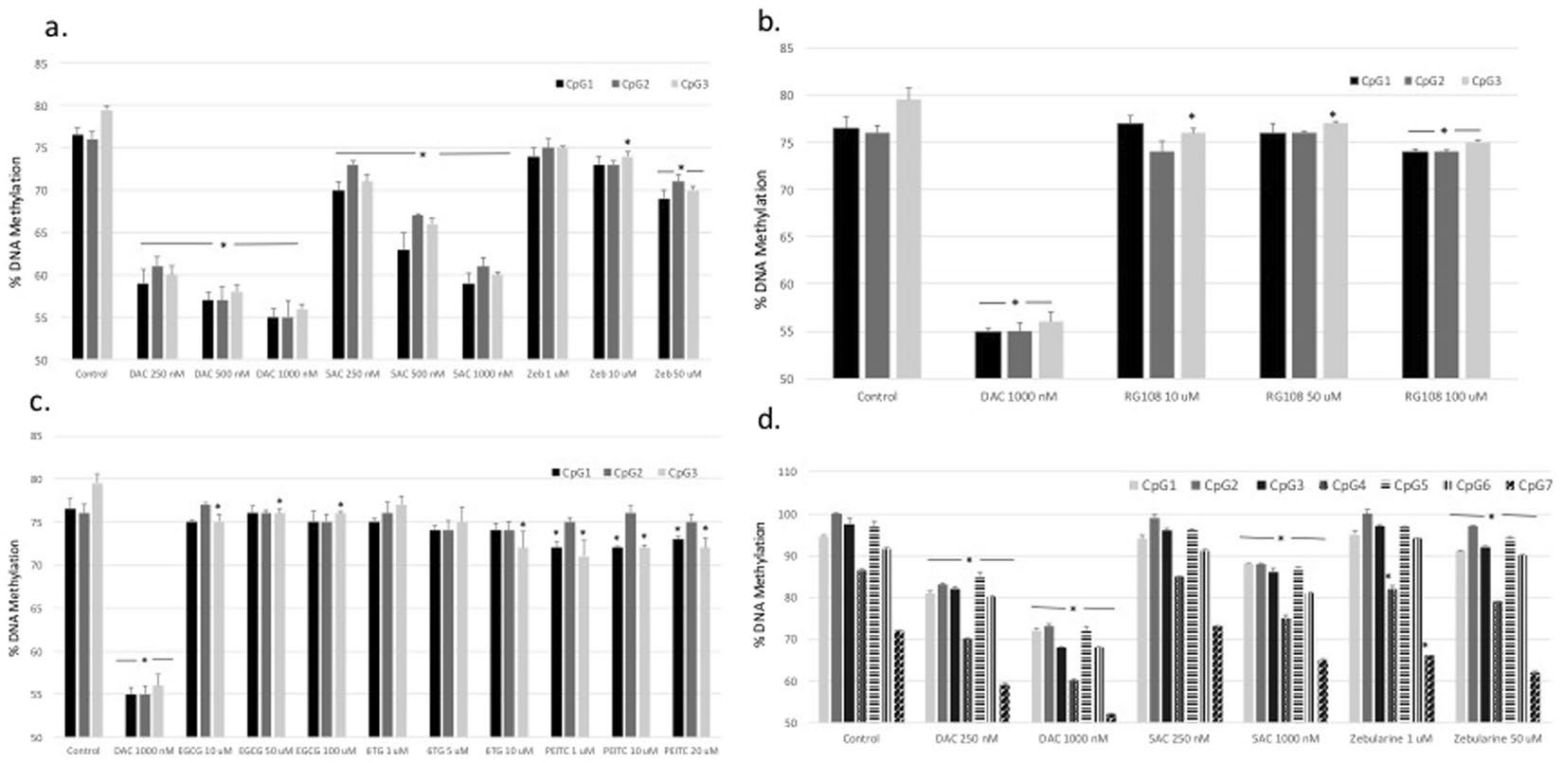
Comparing the DNA hypomethylating activity of DHAs. (**a**) CEM leukemia cells were treated with different concentrations of decitabine (DAC), 5-azacytidine (5AC) and zebularine (Zeb) for 48 h followed by DNA extraction, DNA bisulfite treatment and DNA pyrosequencing of three CpG sites in the LINE-1 sequence (GenBank accession number X58075) as described under methods. (**b**) CEM leukemia cells were treated with different concentrations of decitabine (DAC) or RG-108 for 48 h followed by DNA extraction, DNA bisulfite treatment and DNA pyrosequencing of three CpG sites in the LINE-1 sequence as described under methods. (**c**) CEM leukemia cells were treated with different concentrations of decitabine (DAC), EGCG, 6-thioguanine (6-TG) or PEITC for 48 h followed by DNA extraction, DNA bisulfite treatment and DNA pyrosequencing of three CpG sites in the LINE-1 sequence as described under methods.(**d**) CEM leukemia cells were treated with different concentrations of decitabine (DAC), 5-azacytidine (5AC) and zebularine (Zeb) for 48 h followed by DNA extraction, DNA bisulfite treatment and DNA pyrosequencing of seven CpG sites in the *CDKN2B* (p15) sequence as described under methods. In all experiments, data represent the average of duplicates ± SD. *Indicates significant difference from the corresponding control CpG site at p < 0.05.

The role of DHAs as antitumor agents is well established. We screened the relative potency of eight DHAs in leukemia cells using the gold standard for quantitative DNA methylation, DNA pyrosequencing^18^. The DHAs used were DAC, 5AC, zebularine, the second-generation DNMT inhibitor guadecitabine (SGI-110), the small molecule specific DNMT1 inhibitor RG-108^8^, the thiopurine antileukemic drug 6-thioguanine^19^ and the phytochemicals EGCG and PEITC^11^. CEM leukemia cells were treated with different concentrations of the DHAs for 48 hours followed by global DNA methylation analysis using LINE-1 pyrosequencing of three different CpG sites as described previously^14^. The cytosine nucleoside analogs DAC, 5AC and zebularine demonstrated a dose-dependent methylation reversal with DAC showing the highest potency (Fig. 1a). The second generation hypomethylating agent SGI-110 did not show significant increase in methylation reversal compared to the less metabolically stable DAC at equimolar doses (Supplementary Fig. 1). The activity of the specific DNMT-1 inhibitor RG-108 was significantly less than the nucleoside analogs, even after using 100 times the concentration of DAC, and did not demonstrate dose-dependent methylation reversal (Fig. 1b). Similarly, 6-thioguanine and the phytochemicals EGCG and PEITC did not show dose-dependent methylation reversal and required the use of very high concentrations to induce minimal methylation reversal (Fig. 1c).

Furthermore, gene-specific DNA methylation reversal was tested using DNA pyrosequencing of seven CpG sites within the promoter region of the *CDK2NB* (p15) tumor suppressor gene as previously described^13^. Similar to LINE-1 global methylation assay, the nucleoside analogs demonstrated does-dependent response (Fig. 1d) and were the most potent compared to other DHAs (data not shown). Collectively, the nucleoside analog DAC demonstrated the highest potency in methylation reversal compared to all other DHAs.

### The cytosine nucleoside analogs DHA activate recombinant SIRT6

**Figure 2:**
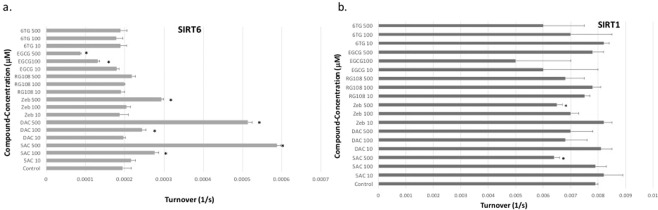
Modulation of the enzymatic activity of SIRT6 and SIRT1 by DHA. Recombinant SIRT6 **(a)** and SIRT1 **(b)** proteins were incubated with different concentrations (10, 100 and 500 μM) of DAC, 5AC, zebularine (Zeb), RG-108, EGCG and 6-thioguanine (6TG) as described under methods. Data represent the mean of 4 replicates ± SD. *Indicates significant difference at p < 0.05.

Previous reports showed that DHAs induce acetylation changes and speculated a mechanism that does not involve direct activation or inhibition of HAT or HDAC^12,20^. To further investigate that, we incubated different human recombinant HDAC sirtuin isoforms (SIRT1, SIRT2, SIRT3, SIRT5 and SIRT6) with DHAs *in vitro* and measured their enzymatic activity as described under methods. The nucleoside analogs DAC, 5AC and zebularine significantly increased the enzymatic activity of SIRT6, while the other DHAs did not induce any significant increase (Fig. 2a). On the other hand, EGCG induced significant decrease in SIRT6 activity. Moreover, both 5AC and zebularine but not DAC decreased the enzymatic activity of SIRT1 (Fig. 2b). The activity of the other sirtuin isoforms (SIRT2, SIRT3 and SIRT5) was not affected by any of the tested DHAs (data not shown). Taken together, the nucleoside analogs DHAs modulated the enzymatic activity of specific sirtuin isoforms and increased the activity of SIRT6.

### Activation of nuclear SIRT6 by the nucleoside analogs DHAs

**Figure 3:**
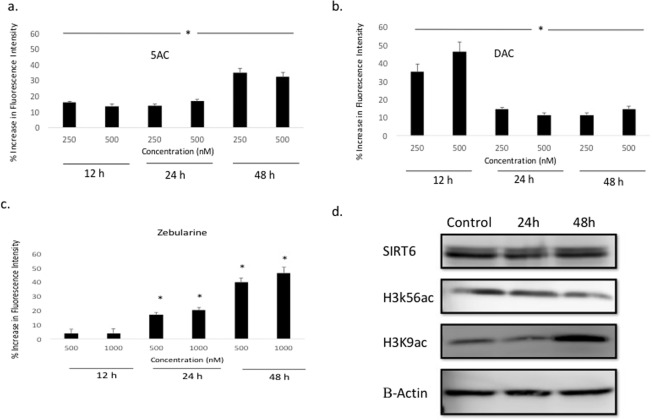
Nucleoside analogs DHAs increase nuclear SIRT6 activity and decrease global expression of acetylated H3K9 and H3K56. U937 leukemia cells were treated with 5AC (**a**), DAC (**b**) and Zebularine (**c**) for 12, 24 and 48 hours followed by nuclear extraction and incubation with a SIRT6 substrate peptide as described under methods. Fluorescence intensity was measured at excitation/emission 480/520 nm and DMSO-treated control was used as a reference to calculate the % increase. Data represent the mean of triplicates ± SD and *indicates significant difference at p < 0.05. (**d**) U937 leukemia cells were treated with 500 nM DAC for 24 and 48 h followed by protein extraction and western blotting for SIRT6, acetylated H3K9 (H3K9ac) and acetylated H3K56 (H3K56ac). Beta-Actin was used as a loading control. The figure represents three different gels full length blots grouped together with dividing lines to indicate that. The full-length blots are included in the supplementary data (Fig. 2).

To further confirm the observed recombinant SIRT6 activation by the nucleoside analogs DHAs, we tested their effect on the activity of nuclear SIRT6 by incubating them with leukemia cells and measuring SIRT6 activity in the nuclear lysates as described under methods. The three drugs 5AC, DAC and zebularine increased SIRT6 activity at different concentrations and time points. Both 5AC and DAC increased SIRT6 activity after 12, 24 and 48 h incubation at different concentrations (Fig. 3a,b, respectively). On the other hand, zebularine increased SIRT6 activity after 24 and 48 h incubation only; albeit at higher concentrations (Fig. 3c). Collectively, these data confirm the activation of SIRT6 by the nucleoside analogs DHAs.

DHAs are known to modulate gene expression and consequently protein expression in tumor cells. Accordingly, we tested the effect of DAC on the mRNA and protein expression of SIRT6 in leukemia cells. No significant changes in SIRT6 mRNA level (data not shown) or protein expression (Fig. 3d) were detected. To further investigate the observed increase in SIRT6 enzymatic activity, the protein expression of global acetylated H3K9 and H3K56 (physiological substrates of SIRT6) was monitored after DAC treatment. A weak decrease in both acetylated H3K9 and H3K56 expression was observed (Fig. 3d) after 24 and 48 h, respectively; consistent with the observed increase in SIRT6 activity and in contrast to stronger decreases observed at specific loci as discussed later. However, acetylated H3K9 showed an increase in expression after 48 h.

### DAC induces genome-wide changes of H3K9 acetylation

**Figure 4:**
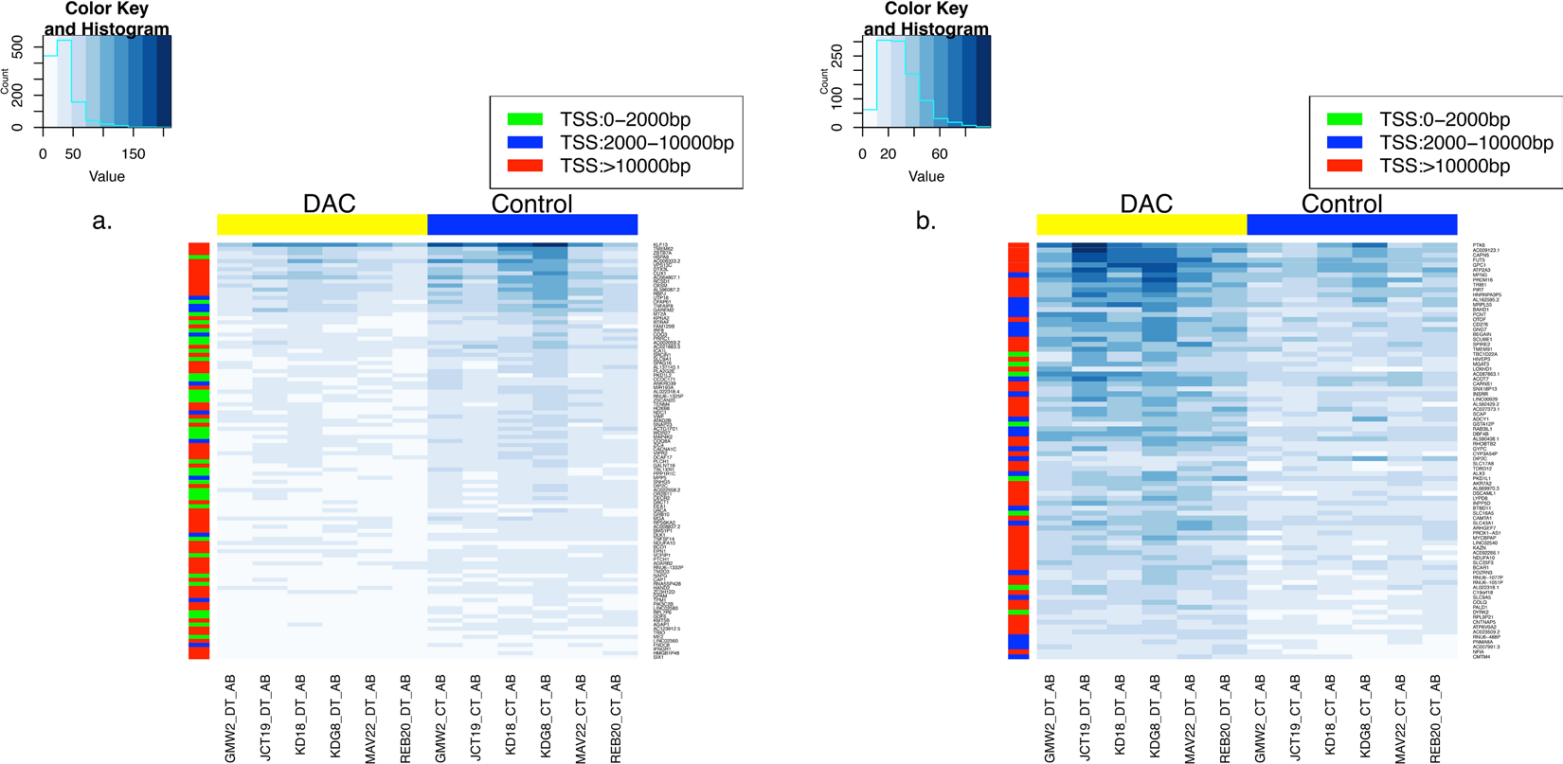
Heatmaps showing the global changes in H3K9 acetylation in AML patient samples treated with DAC. Purified mononuclear cells from bone marrow samples from six AML patients were treated with DAC 500 nM for 72 h and H3K9 acetylation was analyzed by ChIP-Seq as described under methods. The right panel **(a)** shows the panel of the 102 genes showing H3K9 acetylation decrease after DAC treatment and the left panel **(b)** shows the panel of the 85 genes showing H3K9 acetylation increase after DAC treatment. The colored scale at the right of the heatmap shows the position of acetylation change relative to the Transcription Start Site (TSS). The histogram inset at the top shows the value (read count of the peaks) against the count (the number of times those read counts are observed in the heatmap).

**Figure 5:**
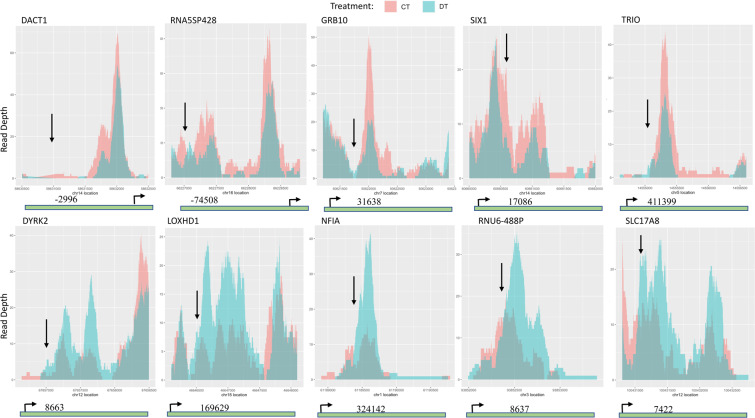
Top 5 genes modulated by DAC treatment. Overlay plots of control and DAC-treated cells showing the chromosomal locations of the peaks of acetylation decrease (upper panel) and acetylation increase (lower panel) of the top 5 genes. The gene map underneath each plot shows the distance from the start of the peak (indicated by the downward arrow) to the TSS (bent arrow). Gene maps are not drawn to scale. DT indicates DAC treatment and CT indicates control cells.

Both H3K9 and H3K56 are known physiological substrates of SIRT6^21^. SIRT6 activation by the nucleoside analogs DHAs is expected to decrease the acetylation of both H3K9 and H3K56 genome-wide. To test that hypothesis, purified bone marrow mononuclear cells from six naïve AML patients were incubated with DAC *in vitro* followed by ChIP-Seq analysis for each patient sample using a validated monoclonal antibody for acetylated H3K9 as described under methods. Data pooling from the six patients showed significant acetylation changes at 187 gene loci after DAC treatment. DAC modulated H3K9 acetylation across the 22 autosomes and at different chromosomal regions including coding exons, introns, distal intergenic regions and promoters. Unexpectedly, both increases and decreases in H3K9 acetylation were observed in the 187 gene loci, where 102/187 genes showed acetylation decrease and 85/187 genes showed acetylation increase. Figures 4a,b show the heatmap for the global decrease and increase in H3K9 acetylation, respectively. Figure 5 shows the overlay of acetylation peaks of the control and DAC-treated cells for the top 5 genes showing H3K9 acetylation decrease and increase. Supplementary Fig. 3a,b show the heatmap for the top 10 genes that showed H3K9 acetylation decrease and increase, respectively. Table 1 and Table 2 show the top 10 genes that showed decrease and increase in H3K9 acetylation, respectively. Supplementary Table 1 and Table 2 provide a detailed list of the global changes in H3K9 acetylation including the gene symbols, gene biotype, fold change in H3K9 acetylation, the position of acetylation change relevant to the transcription start site (TSS), the p-value and the adjusted p-value.

**Table 1:** List of the top 10 genes showing decrease in H3K9 acetylation after DAC treatment.

Gene symbol	Gene biotype	Fold change	Distance from TSS	p-value *10^−4^
DACT1	protein_coding	−2.3	−2966	0.1
GDF6	protein_coding	−2.2	−5791	0.4
GPAM	protein_coding	−2.2	−368976	0.8
GRB10	protein_coding	−2.3	−171761	0.1
HMGB1P48*	unprocessed_pseudogene	−2.2	−826	0.5
IFNGR1	protein_coding	−2.2	92852	0.4
RNA5SP428	rRNA_pseudogene	−2.4	−74508	0.9
SIX1	protein_coding	−2.2	2242	0.4
TPM1	protein_coding	−2.2	25769	0.9
TRIO	protein_coding	−2.4	411399	0.1

*Indicates acetylation decrease within 2000 bp upstream the TSS.

**Table 2:** List of the top 10 genes showing increase in H3K9 acetylation after DAC treatment.

Gene symbol	Gene biotype	Fold change	Distance from TSS	p-value *10^−4^
AC007991.3	antisense	2.4	−63347	0.5
BTBD11	protein_coding	2.2	13588	0.2
CMTM4	protein_coding	2.2	−14006	0.5
DYRK2	protein_coding	2.5	8663	0.2
HIVEP3	protein_coding	2.3	−169724	0.3
LOXHD1	protein_coding	2.4	−10619	0.2
NFIA	protein_coding	2.6	324142	0.4
RNU6-488P	snRNA	2.7	8637	0.3
SLC17A8	protein_coding	2.4	74222	0.8
TDRD12	protein_coding	2.4	40948	0.5

Acetylation decrease in gene promoters (2000 bp upstream the TSS) was observed in 28 genes, while promoter acetylation increase was observed in 6 genes (gray shaded genes in supplementary Tables 1 and 2). CpG islands are CpG rich regions predominantly non-methylated and 70% of gene promoters are associated with a CpG island^22^. CpG islands analysis using the EMBOSS Cpgplot tool of the 34 genes that showed H3K9 acetylation change in their promoter region showed the presence of putative promoter CpG islands in 31 genes (26/31 with acetylation decrease and 5/31 with acetylation increase). The 3 genes that lacked putative promoter CpG islands were *HMGB1P48* (unprocessed pseudogene), *SNAP23* and *C19orf18*.

Gene Ontology (GO) enrichment analysis for the genes with decreased promoter H3K9 acetylation utilizing the PANTHER Overrepresentation Test^23^ and using Fisher’s exact test with FDR correction (p < 0.05) demonstrated their association with several biological processes like cytidine deamination, cytidine to uridine editing, DNA cytosine deamination, DNA demethylation and innate immune response. Pathway analysis by GeneAnalytics geneanalytics.genecards.org linked the changes in H3K9 acetylation induced by DAC to different signaling pathways relevant to hematologic malignancies. *INPP5D, EPN1, GRB10, BCAR1, PTK6, PIK3C2B* and *RPS6KA2* genes were linked to EGF/EGFR signaling pathway, which plays a role in AML^24^. *DLK1, ATP2A3, MFNG, RBPJ, TBL1XR1* genes were linked to NOTCH1 signaling while *DLK1, PTCH1, CUX1, SIX1, RBPJ* were linked to the Wnt/Hedgehog/Notch signaling pathway. Finally, *KPNA2, IRF8, IFNGR1, MT2A, NDC1* were linked to Interferon Gamma signaling. Taken together, DAC induced H3K9 acetylation changes across all the autosomes and in different chromosomal regions and the changes were not limited to acetylation decrease.

## Discussion

In this study, we compared the relative DNA hypomethylating activity of different DHAs and screened their effect on the HDAC sirtuin isoforms SIRT1, SIRT2, SIRT3, SIRT5 and SIRT6. The cytosine nucleoside analogs were more potent than the non-nucleoside DHAs in reversing DNA methylation and increased the enzymatic activity of both recombinant and nuclear SIRT6. The consequences of SIRT6 activation on H3K9 acetylation in primary AML patient samples were monitored using ChIP-Seq analysis. H3K9 acetylation changes were detected in 187 gene loci related to cellular differentiation, proliferation and signaling pathways relevant to leukemia.

DHAs are promising anticancer drug candidates as they induce the expression of epigenetically silenced tumor suppressor genes^25^. In a previous study, the nucleoside analogs were shown to be more effective in reversing DNA methylation compared to the non-nucleoside DHAs like hydralazine, procainamide and EGCG^26^. In this study, we compared the nucleoside analogs to other non-nucleoside DHAs like PEITC, RG-108 and 6-thioguanine and concordantly demonstrated the higher potency of the nucleoside analogs in reversing DNA methylation. Also, DAC demonstrated the highest activity as a DHA compared to the other nucleoside analogs (5AC and zebularine). Contrary to a previous report, RG-108 did not show a dose-dependent methylation reversal and only induced minimal DNA methylation reversal even at very high concentrations^27^.

The pleiotropic biologic effects of the nucleoside analogs DHAs is a challenge in determining the mechanism of action that underpins their clinical efficacy. For instance, DNA methylation reversal of the tumor suppressor *CDKN2B* gene induced by 5AC did not correlate with clinical response in MDS or AML patients, suggesting the possible involvement of mechanisms unrelated to DNA methylation reversal^12,28^. Moreover, both 5AC and DAC induced changes in histone acetylation and methylation by an unknown mechanism^12,29^. In this study, we hypothesized that the nucleoside analogs DHAs modulate the activity of the HDAC sirtuins and demonstrated an increase in the activity of the isoform SIRT6. Unexpectedly, treatment of primary AML cells with DAC induced both increases and decreases in the acetylation of H3K9, the physiological substrate for SIRT6. The unexpected increase in H3K9 acetylation is speculated to be a consequence of the dynamic nature of H3K9 acetylation, where HAT enzymes catalyze the addition of an acetyl group that is removed later by HDAC enzymes. The histone H3K9 is predominantly acetylated by the PCAF/GCN5 class of HAT^30^ and DAC may simultaneously induce differential increase in the enzymatic activity or expression of both the HAT and HDAC enzymes. Additionally, possible activation or increase in expression of other enzymes like the p300 HAT, which also acetylates H3K9 may contribute to the unexpected increase in acetylation^31^. The observed decrease in global H3K9 and H3K56 acetylation by western blotting after DAC treatment is in agreement with the observed SIRT6 activation and the observed H3K9 acetylation decrease by ChIP-Seq. Moreover, the observed increase of acetylated H3K9 after 48 h (Fig. 3d) supports our findings of increased H3K9 acetylation in 85 genes by ChIP-Seq analysis.

H3K9 acetylation on gene promoters is associated with active transcription^32^, accordingly a decrease in gene expression may be expected in the 28 genes that showed acetylation decrease in their promoter region. However, other simultaneous histone modifications should be carefully considered before predicting the impact on gene expression. Our finding that 31/34 genes showing H3K9 promoter acetylation increase or decrease possess a promoter CpG island is concordant with a previous study showing a correlation between H3K9 acetylation and the CpG content^33^. Unfortunately, we could not investigate the reversal of DNA methylation in these promoter regions due to the limited number of cells in the patient samples. On the other hand, the increase in H3K9 acetylation in the promoter region of the genes like *PIRT* and *SCAP* is probably a consequence of DNA demethylation and consequent transcription activation.

The observed changes in H3K9 acetylation within or outside the gene body may also impact gene expression based on the known role of histone acetylation in distal regulatory elements like gene enhancers, silencers and locus control regions in modulating gene expression^34^. Additionally, the changes in H3K9 acetylation in genes involved in leukemia-relevant signaling pathways predict perturbations in these pathways; however, the net effect of these acetylation changes on the signaling pathway and the antitumor effect of DAC requires further investigation.

SIRT6 was shown to be both a tumor suppressor and a promoter depending on the tumor type and cellular context, which adds more complexity in predicting the net biologic effects of its activation by DAC^35^. SIRT6 plays an essential role in maintaining genomic integrity and DNA repair^36^. Recently, CD34^+^ blasts from AML patients demonstrated ongoing DNA damage and SIRT6 overexpression to repair DNA^37^. Depletion of SIRT6 compromised DNA repair in leukemia cells and increased their sensitivity to chemotherapy. Consequently, our findings provide a rationale against the use of DAC or 5AC with SIRT6 inhibitors and their empirical combination with chemotherapeutic agents in AML, especially in patients showing overexpression of SIRT6. On the other hand, our findings will not have a negative impact on the simultaneous or sequential combination of DAC or 5AC with HDAC inhibitors because the currently used HDAC inhibitors like vorinostat, belinostat and romidepsin inhibit only the zinc-dependent HDACs (class I, II and IV) but not the NAD^+^-dependent sirtuins (class III)^38^. Recently, activation of SIRT6 was proven to be a novel therapeutic strategy in different tumors. MDL-800 is a SIRT6 activator that induced global decrease in the acetylation of both H3K56 and H3K9 in human hepatocellular carcinoma and was effective in tumor xenograft model^39^. UBCS039 is another SIRT6 activator that induced autophagy-dependent cell death in solid tumors^40^.

In summary, this study identified a novel biologic target for the nucleoside analogs DHAs. The impact of SIRT6 activation on the overall antitumor effect of DAC requires further investigation due to the complexity and interplay of the posttranslational histone modifications. Our findings shed light on the rational use of the nucleoside analogs DHAs in antitumor combination therapy.

## Materials and Methods

### Cell culture and chemicals

U937 and CEM leukemia cells were obtained from the ATCC (Manassas, VA). Decitabine (DAC), 5-azacytidine (5AC), zebularine, the small molecule specific DNMT1 inhibitor RG-108^8^, the thiopurine antileukemic drug 6-thioguanine^19^, the phytochemicals epigallocatechin gallate (EGCG) and Phenethyl isothiocyanate (PEITC) were all purchased from Millipore Sigma (St. Louis, MO).

### DNA pyrosequencing

Global (LINE-1 methylation) and gene-specific (*CDKN2B/p15*) DNA methylation reversal were quantitated using DNA pyrosequencing as previously described^13,14^.

### Screening the activity of DNA hypomethylating agents using recombinant sirtuins

Deacetylation assay was performed using human recombinant HDAC (SIRT1, SIRT2, SIRT3, SIRT5 and SIRT6) (Active Motif, Carlsbad, CA) and a synthetic peptide substrate for each enzyme. Sirtuin utilizes NAD + as a cofactor to remove the acetyl group from the peptide substrate. This reaction ultimately generates deacetylated peptide, nicotinamide (NAM), and a novel metabolite O-acetyl-ADPribose (AADPR). A typical reaction was performed in 100 mM phosphate buffer pH 7.5 in a total volume of 50 μL. Each reaction contained 500 μM of NAD^+^, 500 μM of substrate, and either 0, 10, 100, or 500 μM of the candidate compound. Control experiments were run at the same time: one contained 500 μM of NAD^+^ alone; the other contained 500 μM of NAD^+^ and 500 μM of candidate compound only. Reactions were initiated with the addition of 10 μM of sirtuin. The reactions were incubated at 37 °C for 2 hours before being quenched by 8 μL of 10% trifluoroacetic acid (TFA). The samples were then injected on an HPLC fitted to a Thermo Scientific Aquasil C18 column. AADPR, NAD^+^ and NAM peaks were resolved using a gradient of 0 to 10% methanol in 20 mM ammonium acetate. Chromatograms were analyzed at 260 nm. Reactions were quantified by integrating areas of peaks corresponding to NAD^+^ and deacetylation product AADPR.

### Quantitation of SIRT6 enzyme activity and expression

A fluorometric assay for measuring the activity of SIRT6 in nuclear extracts was used according to the manufacturer’s instructions (Abcam, Cambridge, MA). Briefly, U937 leukemia cells were treated with DAC, 5AC, Zebularine or DMSO (control) for 12, 24 and 48 hours followed by nuclear extraction and incubation with a SIRT6 substrate peptide coupled to a fluorophore and quencher at its amino terminal and carboxyl terminal, respectively. In absence of SIRT6 deacetylase activity, the fluorescence cannot be emitted because of proximity to the quencher. Upon SIRT6 activation, the substrate peptide will be cleaved by the action of a peptidase enzyme added simultaneously, separating the quencher from the fluorophore and fluorescence will be emitted. The fluorescence intensity is a measure of SIRT6 deacetylase enzyme activity.

SIRT6 protein expression in U937 cells was quantitated after treatment with 500 nM DAC (24 and 48 h) using a monoclonal antibody (Cell Signaling Technology, MA, catalogue # 12486 S) and western blotting as previously described [13]. SIRT6 mRNA expression was quantitated in U937 cells after treatment with DAC (500 nM) for 12, 24 and 48 h by real time RT-PCR as previously described [13]. The following primers for SIRT6 were used: forward primer 5’aggatgtcggtgaattacgc3’ and reverse primer 5’ttggcacattcttccacaaa3’. Quantitation of global H3K9 and H3K56 acetylation was performed using a monoclonal antibody against H3K9ac (Active Motif, catalogue # 61251) and a polyclonal antibody against H3K56ac (Active Motif, catalogue # 39281) and western blotting.

### Patient samples, chromatin Immunoprecipitation (ChIP) and Next Generation Sequencing

Bone marrow mononuclear cells (BMMNC) from six de-identified naïve acute myeloid leukemia (AML) patients were obtained from the tissue bank of Cleveland Clinic. Cells were cultured in RPMI medium supplemented with 10% FBS for 24 hours prior to the experiment in a humidified incubator with 5% carbon dioxide supply. Each patient sample was divided into two groups, one treated with DAC (500 nM) for 72 hours and the other group (control) treated with DMSO. The cell viability was checked before and after drug treatment for each patient sample using the Guava ViaCount reagent (Millipore Sigma) for staining followed by flow cytometry analysis. The viability of all samples was above 90% before and after treatment with DAC (data not shown).

Chromatin crosslinking of the BMMNC was performed for downstream ChIP analysis using a ChIP-validated acetylated-H3K9 (H3K9ac) monoclonal antibody (Active Motif, catalogue # 61251) followed by Next Generation Sequencing (ChIP-Seq). ChIP-Seq libraries were prepared using SMARTer ThruPLEX DNA-seq kit (Takara Bio USA, Mountain View, CA) in the Marshall University Genomic Core (MUGC) facility according to the manufacturer’s instructions. In brief, about 1 to 2 ng fragmented double stranded DNAs were used to construct libraries with dual indices. Libraries were purified and size-selected with AMPure XP beads at 1:1 (v/v) ratio. The resulting libraries were then analyzed on Bioanalyzer 2100 by High Sensitivity DNA chip (Agilent Technologies, Santa Clara, CA). The average range of library amplicons was between 350 bp and 700 bp. Library concentrations were measured by Qubit dsDNA HS Assay kit (ThermoFisher, Waltham, MA). All libraries were pooled prior to sequencing on an Illumina HiSeq. 1500 in the MUGC facility. The dual-indexed single-read Rapid Run was performed with 1×120 cycles at 5 pM final concentration of the library pool. Sequence reads were quality checked using FastQC v0.11.5, and the adapter sequences were removed and low quality (<20) bases at 3-prime ends were trimmed using Trim-galore v0.4.4. Reads were then aligned to the reference human genome (hg38) using Bowtie v2.3.4.1 in an end-to-end alignment mode with default parameters. Alignments with average phred quality score of <10, PCR duplicates and those aligning to blacklisted regions of the genome were filtered out using Samtools v1.4.1. The alignments were converted to bed format using bamtools v2.3.0 and the H3K9ac enriched (peak) regions were identified using diffReps v1.55.6 software with default parameters, using the input samples for background correction. The differentially enriched regions between DAC-treated and control samples were identified using G-test and negative binomial test for Statistical significance for comparisons within each patient sample and for comparisons using all patient samples as replicates, respectively. The peaks regions were annotated with the nearest gene name and its distance to transcription start site, using Bioconductor in R.

### Statistical analyses

Data are represented as the mean ± the standard deviation (SD). Statistical difference between the control and drug-treated samples was calculated using Student’s t-test. p < 0.05 was considered statistically different.

## Supplementary information

Supplementary INFO.
