# Author Correction: Activation of SIRT6 by DNA hypomethylating agents and clinical consequences on combination therapy in leukemia

**DOI:** 10.1038/s41598-020-70622-w

**Published:** 2020-09-22

**Authors:** Hetty E. Carraway, Sridhar A. Malkaram, Yana Cen, Aymen Shatnawi, Jun Fan, Hamdy E. A. Ali, Zakaria Y. Abd Elmageed, Thomm Buttolph, James Denvir, Donald A. Primerano, Tamer E. Fandy

**Affiliations:** 1 Taussig Cancer Institute, Cleveland Clinic, Cleveland, OH USA; 2 Department of Biology, West Virginia State University, Institute, WV USA; 3 Department of Pharmaceutical Sciences, Albany College of Pharmacy, Colchester, VT USA; 4 Department of Pharmaceutical & Administrative Sciences, University of Charleston, Charleston, WV USA; 5 Department of Biomedical Sciences, Marshall University, Huntington, WV USA; 6 Department of Pharmaceutical Sciences, Texas A&M University, Kingsville, TX USA; 7 Department of Neurological Sciences, University of Vermont, Burlington, VT USA; 8 Present Address: Department of Medicinal Chemistry, Virginia Commonwealth University, Richmond, VA 23219 USA

Correction to: *Scientific Reports* 10.1038/s41598-020-67170-8, published online 25 June 2020

The Data Availability statement was omitted in this Article. It is included below:

Data Availability Statement:

The datasets generated during the current study are available in the GEO datasets under Accession number: GSE152804.

