# Supplementary material for: Activation of SIRT6 by DNA hypomethylating agents and clinical consequences on combination therapy in leukemia
Source: Sci Rep. 2020 Jun 25;10:10325. doi: 10.1038/s41598-020-67170-8 (PMC7316973; doi:10.1038/s41598-020-67170-8)
Supplement: Supplementary file 1 — Supplementary INFO. [file 41598_2020_67170_MOESM1_ESM.pdf]

Supplementary Data

Activation of SIRT6 by DNA hypomethylating agents and clinical consequences on combination therapy in leukemia

Hetty E. Carraway[1], Sridhar A. Malkaram[2], Yana Cen[3,*], Aymen Shatnawi[4], Jun Fan[5], Hamdy EA Ali[6], Zakaria Y. Abd Elmageed[6], Thomm Buttolph[7], James Denvir[5], Donald A. Primerano[5], Tamer E. Fandy[4,#]

[1]Taussig Cancer Institute, Cleveland Clinic, Cleveland, OH. [2]Department of Biology, West Virginia State University, Institute, WV. [3]Department of Pharmaceutical Sciences, Albany College of Pharmacy, Colchester, VT. [4]Department of Pharmaceutical & Administrative Sciences, University of Charleston, Charleston, WV. [5]Department of Biomedical Sciences, Marshall University, Huntington, WV. [6]Department of Pharmaceutical Sciences, Texas A&M University, Kingsville, TX [7]Department of Neurological Sciences, University of Vermont, Burlington, VT.

*Current address: Department of Medicinal Chemistry, Virginia Commonwealth University, Richmond, VA 23219

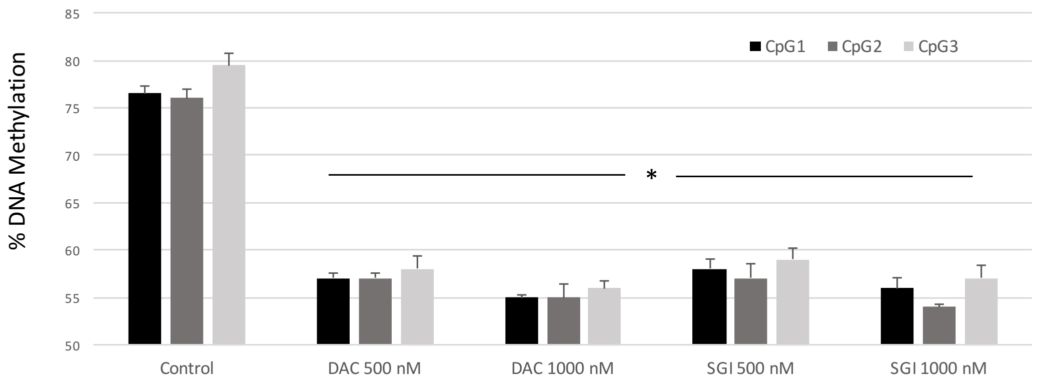

Figure 1. Comparing the DNA hypomethylating activity of DHA. CEM leukemia cells were treated with different concentrations of decitabine (DAC) or guadecitabine (SGI-110) for 48 h followed by DNA extraction, DNA bisulfite treatment and DNA pyrosequencing of three CpG sites in the LINE-1 sequence (GenBank accession number X58075) as described under methods. Data represent the average of duplicates ± SD. * indicates significant difference from control at $p < 0.05$.

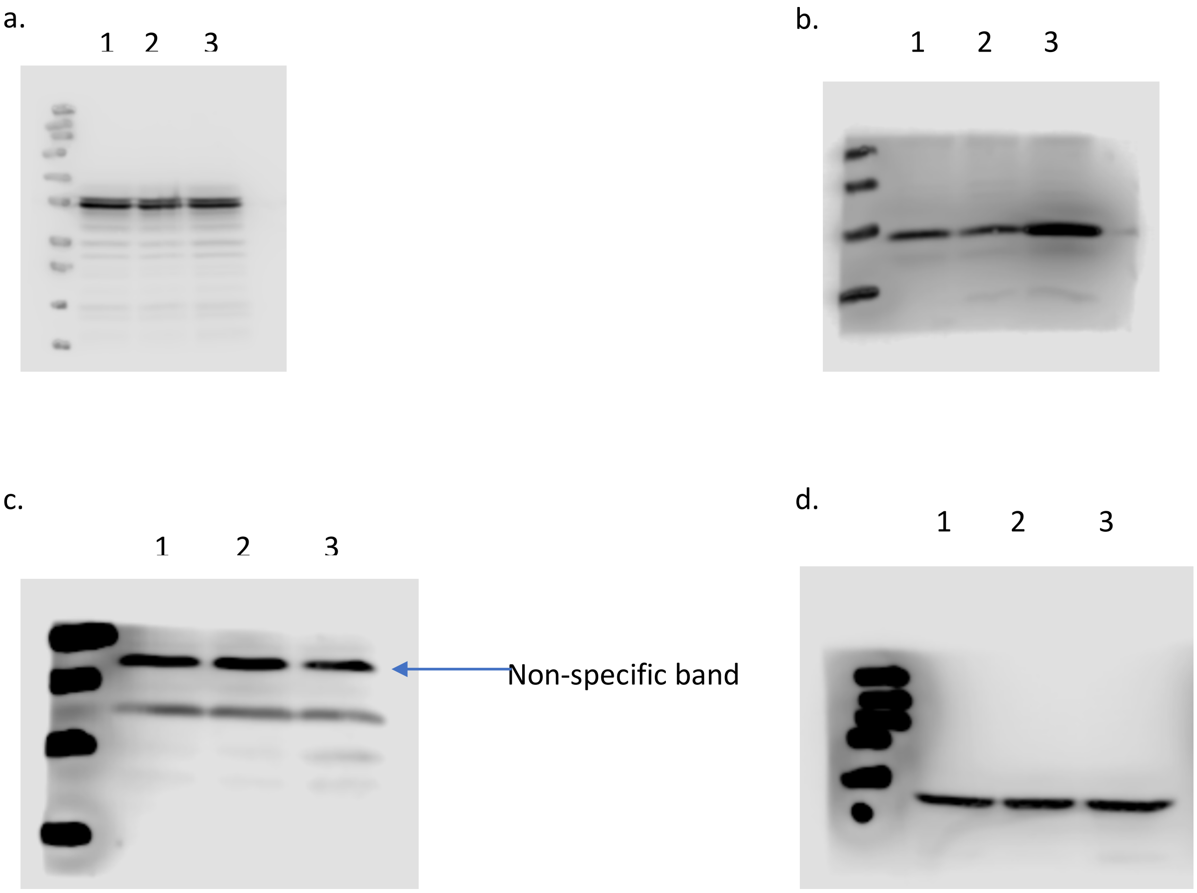

Figure 2. Full length separate western blots of **a.** SIRT6 (37 kDa), **b.** H3K9Ac (17 kDa), **c.** H3K56Ac (17 kDa) and **d.** beta-actin depicted as Figure 3d in the manuscript. Lane 1 represents control cells and lanes 2 and 3 represent U937 cells treated with DAC (500 nM) for 24 and 48 h, respectively.

a.

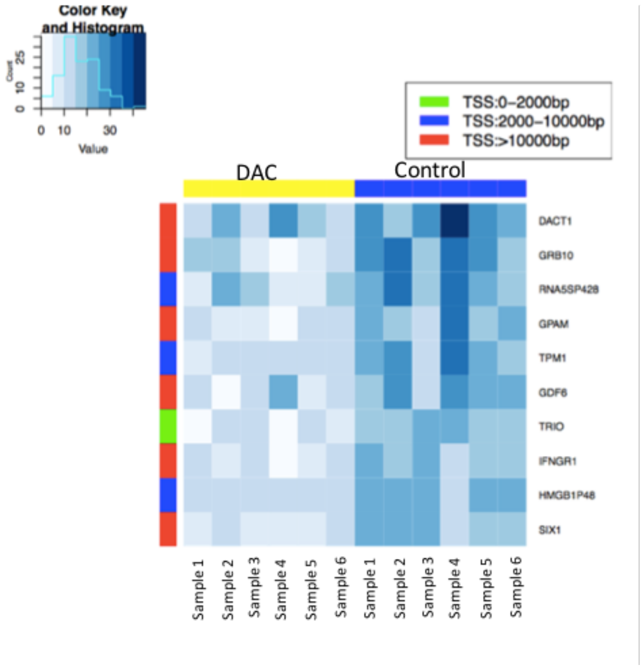

b.

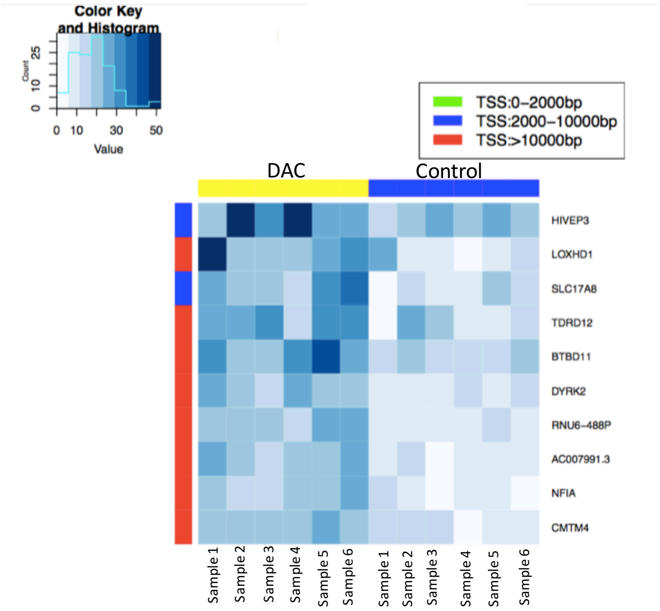

Figure 3. Heatmaps showing the top 10 gene changes in H3K9 acetylation in AML patient samples treated with DAC. Bone marrow samples from six AML patients were treated with DAC 500 nM for 72 h and H3K9 acetylation was analyzed by ChIP-Seq as described under methods. Figure **3a.** shows the top 10 genes showing H3K9 acetylation decrease after DAC treatment and Figure **3b.** shows the top 10 genes showing H3K9 acetylation increase after DAC treatment. The colored scale at the right of the heatmap shows the position of acetylation change relative to the Transcription Start Site (TSS). The histogram inset at the top shows the value (read count of the peaks) against the count (the number of times those read counts are observed in the heatmap).

Table 1. List of genes showing decrease in H3K9 acetylation after DAC treatment. Gray shading indicates acetylation decrease within 2000 bp upstream the TSS.

Gene symbol	Gene biotype	Fold change	Distance from TSS	p-value $*10^{-4}$	Adjusted p-value
AC002059.2	Transcribed unprocessed pseudogene	-1.7	592	0.5	0.20
AC006333.2	antisense	-1.6	-467	0.5	0.20
AC008837.2	processed_pseudogene	-1.6	48121	0.8	0.21
AC021683.5	lincRNA	-1.7	108769	0.09	0.20
AC022558.2	antisense	-1.7	28117	0.7	0.21
AC064807.1	antisense	-1.5	-824	0.9	0.21
AC123912.5	processed_pseudogene	-1.8	-19674	0.8	0.21
ACTG1P21	processed_pseudogene	-1.7	-4163	0.3	0.20
ADARB2	protein_coding	-1.7	-534875	1	0.21
AGAP1	protein_coding	-1.9	558012	0.2	0.20
AL022318.4	protein_coding	-1.6	-62	0.6	0.21
AL137145.1	lincRNA	-1.7	35785	0.2	0.20
AL596087.2	sense_intronic	-1.5	-694	1	0.23
ANKRD39	protein_coding	-1.7	-2794	2	0.30
ATAD2B	protein_coding	-1.6	-91213	0.5	0.20
BCO1	protein_coding	-2.1	52953	0.9	0.20
BMS1P1	transcribed_unprocessed_pseudogene	-1.8	-173	0.4	0.20
CACNA1C	protein_coding	-1.8	468115	0.4	0.20
CAP1	protein_coding	-1.8	-28932	0.6	0.20
CCDC171	protein_coding	-2.0	567915	0.2	0.20
CECR2	protein_coding	-1.8	60852	0.9	0.21
CFAP61	protein_coding	-1.7	-813	0.4	0.20
COQ3	protein_coding	-1.8	-303	0.5	0.20
COQ8A	protein_coding	-1.9	41065	0.5	0.20
CUX1	protein_coding	-1.7	-72603	0.05	0.20
DACT1	protein_coding	-2.3	-2966	0.1	0.20
DCAF17	protein_coding	-1.6	384	0.8	0.21
DIP2C	protein_coding	-1.8	-389667	0.2	0.20
DLK1	protein_coding	-1.8	5696	0.2	0.20
DTX3L	protein_coding	-1.6	-537	0.6	0.20

EEA1	protein_coding	-2.0	-74930	0.8	0.21
EPN1	protein_coding	-1.6	24875	0.9	0.21
FAM129B	protein_coding	-1.9	-23288	0.7	0.21
FNDC8	protein_coding	-1.8	2522	5	0.90
GALNT18	protein_coding	-1.8	-198604	0.5	0.20
GAREM2	protein_coding	-1.6	13510	0.1	0.20
GDF6	protein_coding	-2.2	-5791	0.4	0.20
GPAM	protein_coding	-2.2	-368976	0.8	0.21
GRB10	protein_coding	-2.3	-171761	0.1	0.20
HAND2	protein_coding	-1.9	-13028	0.6	0.20
HMGB1P48	unprocessed_pseudogene	-2.2	-826	0.5	0.20
HOXB8	protein_coding	-1.7	-1738	0.2	0.20
HSPA9	protein_coding	-1.6	-823	0.6	0.20
ICA1L	protein_coding	-1.9	-1584	0.3	0.20
IFNGR1	protein_coding	-2.2	92852	0.4	0.20
IRF8	protein_coding	-1.8	298	1	0.21
KLF13	protein_coding	-1.5	-1354	0.2	0.20
KMT5B	protein_coding	-1.8	-30727	0.7	0.21
KPNA2	protein_coding	-1.9	-518	0.9	0.21
LINC02360	lincRNA	-2.1	-24267	0.7	0.21
LINC02580	lincRNA	-2.1	38010	0.8	0.21
MAP4K2	protein_coding	-1.6	-10940	0.8	0.21
ME2	protein_coding	-2.1	5767	0.2	0.20
MGA	protein_coding	-1.7	39577	0.4	0.20
MIR193A	miRNA	-1.8	-1995	0.8	0.21
MPP5	protein_coding	-1.9	-908	0.1	0.20
MT2A	protein_coding	-1.7	-398	0.5	0.20
NAPG	protein_coding	-2.0	18896	1	0.25
NDC1	protein_coding	-1.8	-559	0.3	0.20
NDUFA10	protein_coding	-1.7	-86001	0.7	0.21
OR2B11	protein_coding	-2.0	10796	0.05	0.20
OXSM	protein_coding	-1.5	184	0.6	0.20
PIK3C2B	protein_coding	-2.0	-37223	0.5	0.20
PKD1L2	polymorphic_pseudogene	-2.1	-119769	0.08	0.20
PLA2G2E	protein_coding	-1.7	-4316	0.6	0.20
PLCH1	protein_coding	-1.7	-203466	0.7	0.21
PPP1R1C	protein_coding	-2.0	35160	0.4	0.20
PRRC1	protein_coding	-1.8	51992	0.6	0.20
PTCH1	protein_coding	-1.8	34544	0.4	0.20

RBPJ	protein_coding	-1.6	156846	0.02	0.20
RCSD1	protein_coding	-1.6	-292	0.4	0.20
RNA5SP428	rRNA_pseudogene	-2.4	-74508	0.9	0.21
RNU6-1325P	snRNA	-1.7	21938	0.5	0.20
RNU6-1332P	snRNA	-1.7	-929	0.9	0.21
RPL7P6	processed_pseudogene	-2.0	-11350	0.2	0.20
RPS6KA2	protein_coding	-1.7	-429050	0.3	0.20
RTRAF	protein_coding	-1.7	-474	0.4	0.2
SIX1	protein_coding	-2.2	2242	0.4	0.2
SLC9A1	protein_coding	-1.8	-11980	0.8	0.21
SNAP23	protein_coding	-2.0	-332	0.4	0.20
SNHG5	processed_transcript	-1.9	-1047	0.4	0.20
SPAG16	protein_coding	-1.7	749422	0.7	0.21
SRCIN1	protein_coding	-2.1	-48529	0.2	0.20
TBL1XR1	protein_coding	-2.0	-32299	0.5	0.20
TENM4	protein_coding	-1.8	-479747	0.5	0.20
TM2D3	protein_coding	-1.8	-34590	0.8	0.21
TMEM62	protein_coding	-1.5	9822	0.7	0.21
TNFAIP8	protein_coding	-1.6	-591	0.1	0.20
TNFSF14	protein_coding	-1.9	113	0.02	0.20
TPM1	protein_coding	-2.2	25769	0.9	0.21
TRIO	protein_coding	-2.4	411399	0.1	0.20
UACA	protein_coding	-1.9	-1792	0.4	0.20
UTP18	protein_coding	-1.6	173	0.8	0.21
VCPIP1	protein_coding	-1.9	-43316	0.1	0.20
VIPR2	protein_coding	-1.8	-60456	0.3	0.20
VPS13C	protein_coding	-1.6	-972	0.7	0.21
VWF	protein_coding	-1.7	-156769	0.2	0.20
WDR27	protein_coding	-1.7	-221766	0.8	0.21
ZBTB7A	protein_coding	-1.6	-1244	1	0.21
ZC3H12D	protein_coding	-1.8	-2560	0.4	0.20
ZIC4	protein_coding	-1.8	-16859	0.7	0.21
ZSCAN20	protein_coding	-1.7	-944	0.1	0.20

Table 2. List of genes showing increase in H3K9 acetylation after DAC treatment. Gray shading indicates acetylation increase within 2000 bp upstream the TSS.

Gene symbol	Gene biotype	Fold change	Distance from TSS	p-value $*10^{-4}$	Adjusted p-value
AC007991.3	antisense	2.4	-63347	0.5	0.20
AC009123.1	antisense	1.6	-52	0.8	0.21
AC023509.2	antisense	1.8	2917	0.4	0.20
AC027373.1	sense_overlapping	1.8	-8887	0.4	0.20
AC087863.1	lincRNA	1.6	-65229	0.8	0.21
AC092266.1	processed_pseudogene	1.9	153042	0.9	0.21
ACOT7	protein_coding	1.7	-108790	0.4	0.20
ADCY1	protein_coding	1.9	84961	0.3	0.20
AKR7A2	protein_coding	1.8	-5645	0.1	0.20
AL022318.1	lincRNA	1.9	14474	0.4	0.20
AL162595.2	antisense	1.8	-33961	0.2	0.20
AL590438.1	lincRNA	1.7	-1095	0.8	0.21
AL592429.2	antisense	1.9	191939	0.1	0.20
AL669970.3	lincRNA	2.1	4719	0.3	0.20
ALX3	protein_coding	1.9	-4699	0.2	0.20
ARHGEF7	protein_coding	1.8	183242	4	0.72
ATP2A3	protein_coding	1.5	-7663	3	0.5
ATP6V0A2	protein_coding	2.0	-2417	0.3	0.20
BAHD1	protein_coding	2.1	17180	0.6	0.20
BCAR1	protein_coding	2.0	-38052	0.1	0.20
BEGAIN	protein_coding	1.8	-4012	0.5	0.20
BTBD11	protein_coding	2.2	13588	0.2	0.20
C19orf18	protein_coding	1.8	-1733	0.9	0.21
CAMTA1	protein_coding	1.9	214277	0.3	0.20
CAPN5	protein_coding	1.8	27460	0.7	0.20
CARNS1	protein_coding	1.8	6833	0.5	0.20
CD276	protein_coding	1.7	28235	1	0.24
CMTM4	protein_coding	2.2	-14006	0.5	0.20
CNTNAP5	protein_coding	1.8	186914	2	0.46
COLQ	protein_coding	1.8	-67950	0.8	0.21

CYP3A54P	processed_pseudogene	2.0	21068	0.7	0.21
DBF4B	protein_coding	1.7	39093	1	0.21
DIP2C	protein_coding	1.7	-133967	0.2	0.20
DSCAML1	protein_coding	2.1	-132924	0.8	0.21
DYRK2	protein_coding	2.5	8663	0.2	0.20
FUT5	protein_coding	1.6	-6439	0.2	0.20
GNG7	protein_coding	1.8	-182908	0.5	0.20
GPC1	protein_coding	1.6	8830	1	0.27
GSTA12P	unprocessed_pseudogene	2.1	-1946	0.6	0.20
GYPC	protein_coding	1.9	17868	0.1	0.20
HIVEP3	protein_coding	2.3	-169724	0.3	0.20
HNRNPA3P5	processed_pseudogene	1.6	-5963	3	0.5
INPP5D	protein_coding	1.7	124434	1	0.25
INSRR	protein_coding	1.7	-12917	3	0.57
KAZN	protein_coding	2.1	959609	0.4	0.20
LINC00929	lincRNA	1.8	9688	0.06	0.20
LINC02540	lincRNA	2.0	-60922	0.9	0.21
LOXHD1	protein_coding	2.4	-10619	0.2	0.20
LYPD8	protein_coding	1.8	5562	0.2	0.20
MFNG	protein_coding	1.6	-7700	0.4	0.20
MGAT3	protein_coding	1.8	34357	0.1	0.20
MRPL55	protein_coding	1.6	-3611	0.4	0.20
MYCBPAP	protein_coding	1.7	18317	0.7	0.21
NFIA	protein_coding	2.6	324142	0.4	0.20
OTOF	protein_coding	1.7	-88397	0.6	0.20
PALD1	protein_coding	2.0	-12020	0.5	0.20
PCNT	protein_coding	1.7	33179	1	0.21
PDZRN3	protein_coding	2.1	-251439	0.3	0.20
PIRT	protein_coding	1.7	-1444	0.4	0.20
PKD1L1	protein_coding	1.7	-58990	0.8	0.21
PNMA8A	protein_coding	2.1	-6062	0.7	0.21
PRDM16	protein_coding	1.7	314133	0.7	0.21

PROX1-AS1	processed_transcript	1.8	-180707	1	0.26
PTK6	protein_coding	1.7	-4269	0.4	0.20
RAB3IL1	protein_coding	1.7	-16668	0.9	0.21
RHOBTB2	protein_coding	2.0	5484	0.6	0.20
RNU6-1051P	snRNA	2.0	-105604	0.7	0.21
RNU6-1077P	snRNA	1.8	-4936	0.9	0.21
RNU6-488P	snRNA	2.7	8637	0.3	0.20
RPL9P21	processed_pseudogene	2.2	20448	2	0.32
SCAP	protein_coding	1.7	-925	0.9	0.21
SCUBE1	protein_coding	1.6	-10987	0.7	0.21
SLC16A5	protein_coding	1.7	8274	0.9	0.21
SLC17A8	protein_coding	2.4	74222	0.8	0.21
SLC35F3	protein_coding	1.9	321968	0.5	0.20
SLC43A1	protein_coding	1.6	-24485	1	0.21
SLC9A5	protein_coding	2.0	818	0.2	0.20
SNX18P13	processed_pseudogene	2.0	313	0.3	0.20
SPIRE2	protein_coding	1.8	28122	2	0.47
TBC1D22A	protein_coding	1.6	171284	3	0.5
TDRD12	protein_coding	2.4	40948	0.5	0.20
TMEM91	protein_coding	1.7	23890	0.5	0.20
TRIB1	protein_coding	1.8	-3120	0.3	0.20
